# Effects of fish and krill oil on gene expression in peripheral blood mononuclear cells and circulating markers of inflammation: a randomised controlled trial

**DOI:** 10.1017/jns.2018.2

**Published:** 2018-03-21

**Authors:** Amanda Rundblad, Kirsten B. Holven, Inge Bruheim, Mari C. Myhrstad, Stine M. Ulven

**Affiliations:** 1 Department of Nursing and Health Promotion, Faculty of Health Sciences, OsloMet – Oslo Metropolitan University, PO Box 4, St. Olavs plass, 0130 Oslo, Norway; 2 Department of Nutrition, Institute for Basic Medical Sciences, University of Oslo, PO Box 1046, Blindern, 0317 Oslo, Norway; 3 National Advisory Unit on Familial Hypercholesterolemia, Department of Endocrinology, Morbid Obesity and Preventive Medicine, Oslo University Hospital, PO Box 4950, Nydalen, 0424 Oslo, Norway; 4 Rimfrost AS, N-6099 Fosnavaag, Norway

**Keywords:** Fish, Krill oil, Marine *n*-3 fatty acids, Peripheral blood mononuclear cells, Glucose, Gene expression, ABCA1, ATP binding cassette A1, ACADVL, acyl-CoA dehydrogenase, very long chain, CD40, cluster of differentiation 40, CPT, carnitine palmitoyltransferase, Ct, cycle threshold, HMGCR, 3-hyroxy-3-methylglutaryl-coenzyme A reductase, HMGCS, 3-hydroxy-3-methylglutaryl-coA synthase, HOSO, high-oleic sunflower oil, ICAM-1, intracellular adhesion molecule-1, PBMC, peripheral blood mononuclear cells, PPARGC1A, PPAR γ coactivator 1A, SCD, steaoryl-CoA desaturase, SLC25A12, solute carrier family 25 member 12, SREBP-1c, sterol-regulating element binding protein 1c, UCP2, uncoupling protein 2, VCAM-1, vascular cell adhesion molecule-1

## Abstract

Marine *n*-3 (omega-3) fatty acids alter gene expression by regulating the activity of transcription factors. Krill oil is a source of marine *n*-3 fatty acids that has been shown to modulate gene expression in animal studies; however, the effect in humans is not known. Hence, we aimed to compare the effect of intake of krill oil, lean and fatty fish with a similar content of *n*-3 fatty acids, and high-oleic sunflower oil (HOSO) with added astaxanthin on the expression of genes involved in glucose and lipid metabolism and inflammation in peripheral blood mononuclear cells (PBMC) as well as circulating inflammatory markers. In an 8-week trial, healthy men and women aged 18–70 years with fasting TAG of 1·3–4·0 mmol/l were randomised to receive krill oil capsules (*n* 12), HOSO capsules (*n* 12) or lean and fatty fish (*n* 12). The weekly intakes of marine *n*-3 fatty acids from the interventions were 4654, 0 and 4103 mg, respectively. The mRNA expression of four genes, PPAR γ coactivator 1A (*PPARGC1A*), steaoryl-CoA desaturase (*SCD*), ATP binding cassette A1 (*ABCA1*) and cluster of differentiation 40 (*CD40*), were differently altered by the interventions. Furthermore, within-group analyses revealed that krill oil down-regulated the mRNA expression of thirteen genes, including genes involved in glucose and cholesterol metabolism and β-oxidation. Fish altered the mRNA expression of four genes and HOSO down-regulated sixteen genes, including several inflammation-related genes. There were no differences between the groups in circulating inflammatory markers after the intervention. In conclusion, the intake of krill oil and HOSO with added astaxanthin alter the PBMC mRNA expression of more genes than the intake of fish.

Intake of fish and fish oil is shown to reduce CVD risk, and the marine omega-3 (*n*-3) fatty acids EPA (20 : 5*n*-3) and DHA (22 : 6*n*-3) are involved in mediating this beneficial effect^(^^1^^–^^5^^)^. Krill oil is an alternative source of marine *n*-3 fatty acids that has gained interest because fish and fish oil are limited resources. Unlike *n*-3 fatty acids in fish oil that are stored in TAG, in krill oil up to 65 % are stored as phospholipids^(^^6^^)^. It is suggested that this renders the *n*-3 fatty acids more bioavailable and bioactive than in fish oil^(^^7^^)^. In addition to *n*-3 fatty acids, krill oil contains astaxanthin, an antioxidant with potential health-beneficial effects^(^^6^^,^^8^^,^^9^^)^.

The benefit of *n*-3 fatty acids on CVD risk is mainly attributed to the TAG-reducing effect. Additionally, intake of both lean and fatty fish has been shown to reduce TAG levels, and because lean fish contain relatively low amounts of *n*-3 fatty acids, other nutrients might be involved^(^^10^^–^^13^^)^. Krill oil may reduce TAG, especially in subjects with elevated TAG, although more human studies are needed to clarify if the effects of krill oil on lipid metabolism differ from the effects of fish and fish oil^(^^7^^,^^14^^–^^19^^)^. Another beneficial effect of *n*-3 fatty acids is their ability to reduce levels of circulating inflammatory markers, such as TNFα, IL-1β, IL-6 and IL-8 and intracellular adhesion molecule-1 (ICAM-1) and vascular cell adhesion molecule-1 (VCAM-1) in various patient groups; however, the effect in healthy humans and individuals with a high CVD risk is less clear^(^^20^^,^^21^^)^. A recent meta-analysis concluded that *n*-3 supplementation reduces C-reactive protein, but has no significant effect on TNFα, ICAM-1 and VCAM-1^(^^22^^)^. Compared with fish oil, intake of fish seems to have a more marked effect on circulating inflammatory markers^(^^23^^)^. Knowledge about krill oil effects on inflammation in humans is very limited, and in a previous krill oil study in our group, no effect on inflammatory markers was observed^(^^17^^)^.

Although the mechanisms underlying the beneficial effects of *n*-3 fatty acids are not fully understood, they include modification of lipid rafts, alteration of membrane fatty acid composition and modulation of gene expression^(^^20^^,^^24^^)^. Marine *n*-3 fatty acids may modulate the expression of genes involved in inflammation by activating PPAR^(^^25^^)^. These nuclear receptors are ligand-inducible transcription factors that inhibit the activation of NF-κB, a major transcriptional factor regulating the transcription of inflammation genes. Additionally, *n*-3 fatty acids act anti-inflammatory by binding directly to the G-protein-coupled receptor 120 (GPR120) and thereby inhibit NF-κB activation^(^^20^^)^. The TAG-lowering effect of fish oil has also been suggested to be mediated by activation of PPARα that induces the expression of genes involved in β-oxidation, making fatty acids less available for TAG synthesis^(^^26^^)^. Furthermore, *n*-3 fatty acids reduce the nuclear levels of sterol-regulating element binding protein 1c (SREBP-1c) and thereby reduce the transcription of genes in *de novo* lipogenesis^(^^27^^)^.

Peripheral blood mononuclear cells (PBMC) include lymphocytes and monocytes and play a central role in inflammation, and thus the development of CVD^(^^28^^)^. Because they are circulating cells, PBMC are exposed to nutrients, metabolites and peripheral tissues such as liver and adipose tissue. Hence, they may reflect whole-body health status^(^^29^^)^. PBMC are readily accessible and PBMC gene expression is suggested as a model to investigate the effect of interventions on lipid metabolism and inflammation^(^^30^^)^. Several studies have investigated the effect of intake of fish oil^(^^31^^–^^33^^)^ and fatty or lean fish^(^^11^^,^^34^^)^ on PBMC gene expression; however, the effect of a combination of lean and fatty fish is less studied. The effect of krill oil on gene expression in humans is not known; however, when given in equal doses, krill oil modulated the expression of more genes than fish oil in a study of the hepatic transcriptome in mice, indicating that *n*-3 fatty acids from krill oil might be more bioactive^(^^35^^)^.

We recently performed a randomised controlled study where we compared the effects of intake of krill oil capsules, lean and fatty fish with a similar content of *n*-3 fatty acids, and high-oleic sunflower oil (HOSO) capsules with added astaxanthin. We showed that intake of krill oil reduced fasting glucose and that intake of lean and fatty fish increased vitamin D levels, while plasma levels of marine *n*-3 fatty acids increased in both groups receiving *n*-3 fatty acids^(^^18^^)^. The aim of the present study was to further investigate the effect on PBMC expression of genes related to lipid and glucose metabolism and inflammation. Additionally, we wanted to analyse the effect of selected circulating markers of inflammation and endothelial dysfunction.

## Methods

### Subjects and study design

Healthy subjects aged 18–70 years were recruited from Skedsmo in the Akershus county, Norway, primarily by invitations by post. We included subjects with fasting serum TAG between 1·3 and 4·0 mmol/l, a stable weight (±5 %) over the past 3 months, BMI between 18·5 and 35 kg/m^2^ and high-sensitivity C-reactive protein <10 mg/l. In brief, exclusion criteria were cancer or CVD in the past 6 months or any chronic disease including diabetes type 1 or 2, hypertension (>160/100 mm Hg), total cholesterol >7·8 mmol/l and regular intake of fatty fish more than once every week. Initially, the inclusion criterion for fasting TAG was 1·7–4·0 mmol/l; however, the lower limit was changed in order to include more participants. Consumption of *n*-3 supplements was not allowed during the study, and there was a 4-week washout period prior to inclusion for participants with a regular intake. Consumption of fatty fish was not allowed and the consumption of lean fish was restricted to the maximum of one meal per week from the screening visit and throughout the intervention period. This also applied to participants in the fish group until randomisation. Subjects were instructed to maintain their habitual diet and level of physical activity.

The present study was an 8-week randomised controlled three-armed parallel-group trial with an allocation ratio of 1:1:1. The fish group received three weekly fish meals, one dinner meal with cod, one dinner meal with salmon and one bread spread containing mackerel. Participants in the krill group and the control group were instructed to take eight capsules per d containing 4 g/d of krill oil (RIMFROST Sublime®) or HOSO. The weekly intake of marine *n*-3 fatty acids were 4103, 4654 and 0 mg in the fish group, krill group and control group, respectively. A total of thirty-six subjects, twelve in each intervention group, completed the 8-week intervention that lasted from October 2015 to November 2016. The control group and the krill group were blinded for the investigators and participants. This was achieved by delivering the capsules in identical containers and by adding astaxanthin to the HOSO in an equal concentration as in the krill oil (982 parts per million) to obtain a similar colour. Compliance, estimated by capsule count in the krill group and the control group and a compliance checklist in the fish group, was 97, 97 and 100 %, respectively. The recruitment of subjects, eligibility criteria, description of study products and the design of the study have previously been described in detail^(^^18^^)^.

### Ethics statement

This study was conducted according to the guidelines laid down in the Declaration of Helsinki and all procedures involving human subjects were approved by the Regional Ethics Committee for Medical Research in South East Norway (2015/706/REK sør-øst C). Written informed consent was obtained from all subjects. The study was registered at http://www.clinicaltrials.gov (ClinicalTrials.gov identifier: NCT02568228).

### Blood sampling and isolation of peripheral blood mononuclear cells

Participants were instructed to avoid vigorous physical activity and alcohol consumption and to eat a low-fat dinner meal the day before blood sampling. Fasting blood samples were drawn at baseline and at the end of study. Whole blood was collected in EDTA tubes and was kept at room temperature for a maximum of 48 h before leucocyte count at a routine laboratory (Fürst Medical Laboratory, Oslo, Norway). Serum was obtained from silica gel tubes (Becton Dickinson Vacutainer Systems) that were kept at room temperature for at least 30 min until centrifugation (1500 ***g***, 15 min). PBMC were isolated using BD Vacutainer Cell Preparation Tubes according to the manufacturer's instructions (Becton, Dickinson) and cell pellets were stored at −80°C. Routine laboratory analyses, including fasting serum TAG, glucose, cholesterol and vitamin D, were measured at a standard clinical routine laboratory (Fürst Medical Laboratory, Oslo, Norway). Plasma fatty acid analysis was performed at a certified chemical analysis laboratory (Vitas Analytical Service, Oslo, Norway), as previously described^(^^18^^)^.

### Isolation of RNA, complementary DNA synthesis and quantitative real-time PCR

Total RNA, including microRNA, was isolated using the miRNeasy kit using the automated protocol for the QIAcube according to the manufacturer's instructions (Qiagen). The quantity and quality of the RNA were analysed using a Nanodrop ND-1000 Spectrophotometer (Thermo Fisher Scientific) and the Agilent RNA 6000 Nano kit using the Agilent 2100 Bioanalyzer (Agilent Technologies). All samples had an RNA integrity number (RIN) > 8. RNA was reverse transcribed to complementary DNA using a high-capacity complementary DNA reverse transcription kit (Applied Biosystems). Quantitative real-time PCR (qPCR) was applied for the analysis of gene expression using custom-designed TaqMan Low-Density Array (LDA) Microfluidic Cards on an ABI PRISM 7900HT (Applied Biosystems). A total of forty-four target genes were selected based on studies showing that the expression of genes involved in inflammation and lipid and glucose metabolism is altered in response to fish consumption and supplementation with krill oil and fish oil^(^^11^^,^^34^^–^^40^^)^. In quality assessment, four genes were excluded from analysis (3-hydroxy-3-methylglutaryl-coA synthase 2 (*HMGCS2*), MLX-interacting protein-like (*MLXIPL*), *IL6* and C-X-C motif chemokine ligand 8 (*CXCL8*)). The relative change in gene expression was calculated using the ΔΔ cycle threshold (Ct) method^(^^41^^)^. Ct values of each gene were normalised to the reference gene, TATA binding protein (*TBP*) (Ct_reference_ − Ct_target_ = ΔCt) and the relative change was calculated from baseline to the end of the study (ΔCt_end of study_ − ΔCt_baseline_ = ΔΔCt), expressed as log ratio. *TBP* was chosen as the reference gene based on lower between-sample variation than glucuronidase β (*GUSB*), *18SRNA* and filamin B (*FLNB*).

### ELISA

Serum levels of TNF-α were analysed with Quantikine high-sensitivity ELISA kits (R&D Systems). Levels of ICAM-1 and VCAM-1 were analysed with DuoSet ELISA (R&D Systems) and Quantikine ELISA (R&D Systems), respectively. The level of NO metabolites (NOx) in serum was measured with Parameter ELISA (R&D Systems). All assays were performed according to the manufacturer's instructions.

### Statistical analysis

The power calculation has been described previously^(^^18^^)^. Briefly, we expected individuals with a TAG level of 2·21 (sd 0·59) mmol/l to have a 20 % decrease in the primary end point, fasting TAG, after intake of 800 mg EPA + DHA per d from krill oil. A total of ninety-six participants were required given a 10 % drop-out rate, a power of 80 % and a significance level of 5 %; however, due to challenges in participant recruitment we randomised only forty participants.

Changes in mRNA levels (ΔCt) and circulating inflammatory markers from baseline to the end of the study were tested with a paired *t* test. Differences between the three intervention groups in log ratio (ΔΔCt) and change in circulating inflammatory markers were tested with one-way ANOVA. In the case of significant findings, *post hoc* comparisons were performed with the pairwise *t* test with Bonferroni-corrected *P* values. Correlations were tested with Pearson's correlation. Because of the exploratory design of this study, results were not adjusted for multiple comparisons. *P* values ≤0·05 are considered significant. All statistical analyses were performed in R^(^^42^^)^ and were per protocol.

## Results

### Baseline characteristics

A total of thirty-six subjects, twelve in each intervention group, completed the study and samples from all thirty-six participants were used for the analysis of gene expression and circulating inflammatory markers. Baseline characteristics of the three intervention groups and a flowchart illustrating the number of participants screened, randomised and lost to follow-up have been reported previously^(^^18^^)^. Briefly, the participants were aged on average 56 (sd 8) years, they had elevated fasting TAG (1·7 (sd 0·63) mmol/l) elevated LDL-cholesterol (3·8 (sd 0·97) mmol/l) and had an average BMI of 27·3 (sd 3·7) kg/m^2^. There were 50 % men and 50 % women in each group.

### Effect of the interventions on the expression levels of genes

**Fig. 1.**
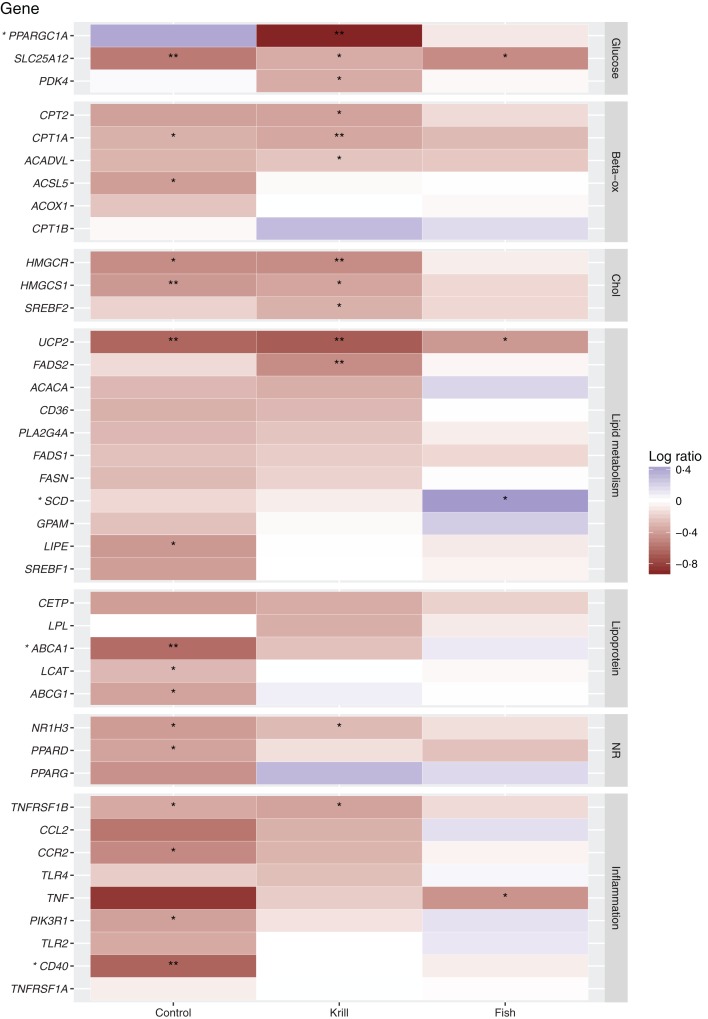
Gene expression heatmap, showing the log ratio gene expression change (ΔΔ cycle threshold; ΔΔCt). Red indicates a reduced mRNA gene expression and blue indicates an increase, with increasing colour intensity for larger effects. Genes with a significantly different change between the three intervention groups, tested with one-way ANOVA, are indicated with asterisks by the gene names. Significant change from baseline to end of study within each group: * *P* ≤ 0·05, ** *P* ≤ 0·01 (paired *t* test). Beta-ox, β-oxidation; chol, cholesterol; NR, nuclear receptors. For gene names, see the abbreviations list and the text.

The change in gene expression was significantly different between the three groups for four genes: PPAR γ coactivator 1A (*PPARGC1A*; *P* = 0·01), steaoryl-CoA desaturase (*SCD*; *P* = 0·05), ATP binding cassette A1 (*ABCA1*; *P* = 0·03) and cluster of differentiation 40 (*CD40*; *P* = 0·04) (Fig. 1 and Supplementary Appendix S1). *Post hoc* pairwise comparisons revealed that the increased expression of *PPARGC1A* in the control group was significantly different from the decreased expression in the krill group (*P* = 0·01) and that the decreased expression in *ABCA1* in the control group was significantly different from the small increase in the fish group (*P* = 0·02). The change in mRNA expression of *CD40* and *SCD* did not differ between any pair of groups in *post hoc* analyses.

### Down-regulation of mRNA expression of genes by krill oil

Within-group analyses showed that intake of krill oil significantly down-regulated the mRNA expression level of thirteen of the forty genes that we analysed in PBMC (Fig. 1). These included three genes encoding proteins involved in glucose metabolism (*PPARGC1A* (*P* = 0·008), pyruvate dehydrogenase kinase 4 (*PDK4*; *P* = 0·04) and solute carrier family 25 member 12 (*SLC25A12*; *P* = 0·01)), three genes encoding proteins involved in cholesterol metabolism (3-hyroxy-3-methylglutaryl-coenzyme A reductase (*HMGCR*; *P* = 0·001), *HMGCS1* (*P* = 0·02) and sterol regulatory element binding factor 2 (*SREBF2*; *P* = 0·01)) as well as three genes encoding proteins involved in fatty acid β-oxidation (acyl-CoA dehydrogenase, very long chain (*ACADVL*; *P* = 0·02) and carnitine palmitoyltransferase 1a and 2 (*CPT1a*; *P* = 0·009 and *CPT2*; *P* = 0·01)). Finally, the mRNA expression of various genes encoding nuclear receptors and proteins involved in lipid metabolism and inflammation was reduced by krill oil: nuclear receptor subfamily 1 group H member 3, commonly known as LXRα (*NR1H3*; *P* = 0·02), fatty acid desaturase 2 (*FADS2*; *P* = 0·006), uncoupling protein 2 (*UCP2*; *P* = 0·002) and TNF receptor superfamily 1B (*TNFRSF1B*; *P* = 0·01).

### Effect of lean and fatty fish on mRNA expression of genes

Intake of lean and fatty fish significantly altered the mRNA expression of four genes (Fig. 1). Of these, the mRNA expression of *SCD* was up-regulated (*P* = 0·05), and the expression levels of *SLC25A12* (*P* = 0·05), *UCP2* (*P* = 0·05) and *TNF* (*P* = 0·03) were down-regulated.

### Down-regulation of mRNA expression of genes by high-oleic sunflower oil with added astaxanthin

Intake of HOSO with added astaxanthin significantly reduced the mRNA expression of sixteen genes from baseline to the end of the study (Fig. 1). These included *NR1H3* (*LXRα*; *P* = 0·007), and the LXR target genes *ABCA1* (*P* = 0·005) and *ABCG1* (*P* = 0·02) and four inflammation-related genes: *CD40* (*P* = 0·001), C-C chemokine receptor 2 (*CCR2*; *P* = 0·02), *TNFRSF1B* (*P* = 0·04) and phosphatidylinositol 3-kinase regulatory subunit 1 (*PIK3R1*; *P* = 0·01). Finally, HOSO down-regulated various genes involved in lipid and glucose metabolism: hormone-sensitive lipase (*LIPE*; *P* = 0·04), *UCP2* (*P* = 0·002), *HMGCR* (*P* = 0·01), *HMGCS1* (*P* = 0·005), *SLC25A12* (*P* = 0·008), *CPT1A* (*P* = 0·04), acyl-CoA synthetase long chain family member 5 (*ACSL5*; *P* = 0·04), lecithin-cholesterol acyltransferase (*LCAT*; *P* = 0·05) and PPAR-δ (*PPARD*; *P* = 0·05).

### Correlations

**Fig. 2.**
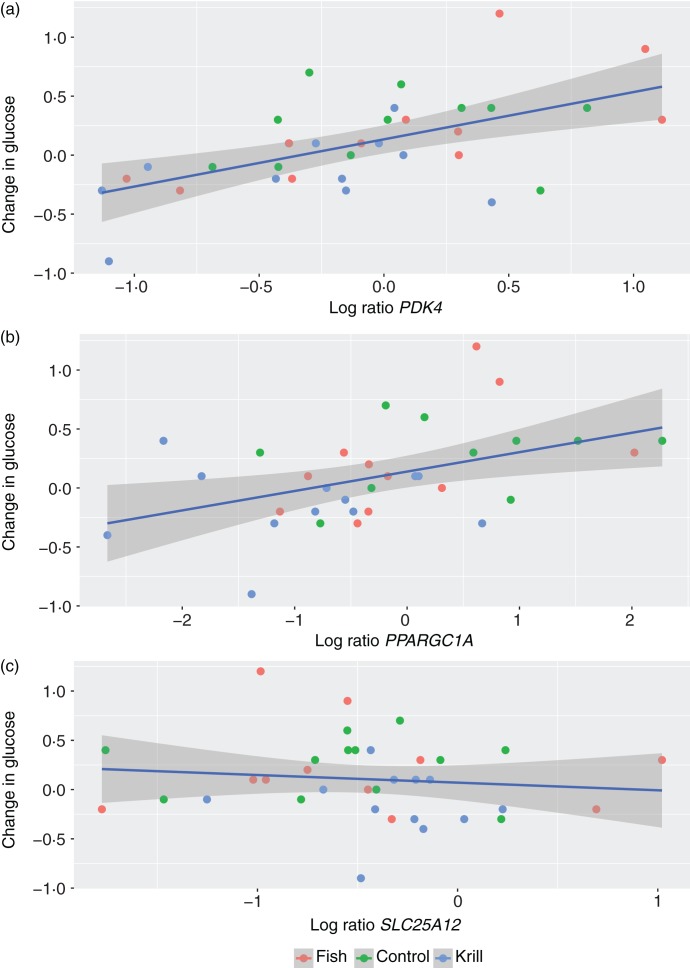
Scatter plot of change in fasting glucose (mmol/l) and log ratio gene expression change (ΔΔ cycle threshold; ΔΔCt) of (a) pyruvate dehydrogenase kinase 4 (*PDK4*) (*P* = 0·0005; *r* 0·6), (b) PPAR γ coactivator 1A (*PPARGC1A*) (*P* = 0·01; *r* 0·4) and (c) solute carrier family 25 member 12 (*SLC25A12*) (*P* = 0·5; *r* −0·1).

We have previously reported that fasting glucose decreased in the krill group, vitamin D levels increased in the fish group and plasma *n*-3 levels increased in both the krill and fish groups^(^^18^^)^; hence, we wanted to correlate these changes with the change in gene expression of glucose metabolism-related genes, inflammation-related genes and all the genes analysed, respectively. First, we found a positive and significant correlation between the change in fasting glucose and the change in gene expression of *PDK4* and *PPAGC1A*, but not *SLC25A12* (Fig. 2). Furthermore, we found a negative and significant correlation between the change in vitamin D levels and the change in *TNF* expression (*P* = 0·05; *r* −0·3). No other inflammation-related genes correlated with the change in vitamin D levels (Supplementary Appendix S2). Finally, we found that the change in gene expression of *PPARGC1A* correlated negatively (*P* = 0·006; *r* −0·5) and *SREBF1* correlated positively (*P* = 0·04; *r* 0·4) with the change in plasma total *n*-3 fatty acids. However, the change in plasma *n*-3 fatty acids did not correlate with the change in any of the other thirty-eight genes analysed (Supplementary Appendix S3).

### Effects on circulating markers of inflammation and endothelial dysfunction

To further investigate the effect on inflammation and endothelial dysfunction, circulating levels of TNF-α, VCAM-1, ICAM-1 and NO (NOx) were analysed. There were no differences between the groups in any of the circulating markers; however, ICAM-1 was significantly increased within the fish group (*P* = 0·02) (Table 1).

**Table 1.** Levels of circulating inflammatory markers at baseline and at the end of the study* (Mean values with their standard errors)

	Control group					Krill group					Fish group					
	Baseline		End of study			Baseline		End of study			Baseline		End of study			
	Mean	sem	Mean	sem	*P*†	Mean	sem	Mean	sem	*P*†	Mean	sem	Mean	sem	*P*†	ANOVA *P*
TNF-α (pg/ml)	1·04	0·05	1·09	0·08	0·35	0·89	0·04	0·95	0·06	0·30	0·89	0·05	0·89	0·06	0·90	0·67
VCAM (ng/ml)	563·3	39·3	545·1	33·0	0·66	551·9	48·5	493·5	44·1	0·35	414·0	31·3	475·5	53·4	0·12	0·20
ICAM (ng/ml)	108·7	13·6	109·1	12·2	0·89	95·3	7·0	101·1	10·6	0·23	102·6	8·3	109·3	8·9	0·02	0·41
NOx (μmol/l)	25·5	2·9	25·0	4·2	0·92	18·6	2·1	21·0	2·5	0·06	16·4	1·7	19·5	2·5	0·11	0·69

VCAM-1, vascular cell adhesion molecule-1; ICAM-1, intracellular adhesion molecule-1; NOx, nitric oxide.

* The differences within groups were tested by paired *t* test, while the differences between the groups were tested with one-way ANOVA.

† *P* value obtained from paired *t* test for change within the group.

## Discussion

In the present study, we investigated the effect on PBMC gene expression after intake of krill oil, lean and fatty fish and HOSO with added astaxanthin. We found that the expression of four genes, *PPARGC1A*, *SCD*, *ABCA1* and *CD40*, was differently affected by the three interventions. Furthermore, we found that intake of krill oil and HOSO with added astaxanthin altered the expression of more genes than the intake of fish. The thirteen genes with a down-regulated expression within the krill group included genes involved in glucose and cholesterol metabolism and β-oxidation. The sixteen genes with a down-regulated expression within the control group included genes encoding proteins involved in inflammation and cholesterol efflux.

The mRNA expression levels of *PPARGC1A*, *PDK4* and *SLC25A12* were down-regulated within the krill group. In addition, the change in *PPARGC1A* expression was significantly different between the three intervention groups and pairwise comparisons revealed that the decrease in the krill group differed from the increased expression in the control group. PPARGC1A is a major regulator of gluconeogenesis, and a down-regulation of this gene would thus reduce expression of other genes in gluconeogenesis. Indeed, PPARGC1A is suggested as a drug target for the treatment of type 2 diabetes^(^^43^^)^. PDK4 inhibits pyruvate dehydrogenase; hence, a down-regulation of *PDK4* shifts the metabolism toward glucose utilisation. SLC25A12 transports aspartate from the mitochondria to cytosol in the exchange for glutamate. Aspartate may then be used as a precursor for oxaloacetate in gluconeogenesis^(^^44^^)^. In summary, the down-regulation of these three genes may induce a shift toward glucose utilisation and away from gluconeogenesis. We have previously reported that intake of krill oil reduced fasting glucose in the same intervention^(^^18^^)^, and here we show that the change in fasting glucose correlates with the change in gene expression of *PDK4* and *PPARGC1A*. The reduction in fasting glucose observed after krill oil intake may thus be partly mediated through down-regulation of the expression of these two genes. Moreover, the change in *PPARGC1A* expression correlated with the change in plasma *n*-3 fatty acids, indicating that it might be the increased intake of *n*-3 fatty acids in the krill group that mediated this change. In line with our findings, Burri *et al.*^(^^35^^)^ previously showed by pathway analysis of the hepatic transcriptome in mice that krill oil down-regulated the gluconeogenesis pathway, and, interestingly, the expressions of *Ppargc1a* and *Slc25a12* were reduced.

Sterol regulatory element binding protein 2 (SREBP2), the protein product of *SREBF2*, is the major transcription factor regulating the expression of genes involved in cholesterol metabolism, including *HMGCR* and *HMGCS1*. These three genes were down-regulated after the intake of krill oil. Correspondingly, krill oil and krill powder have previously been shown to reduce the hepatic expression of *Srebf2*, *Hmgcr* and *Hmgcs1* in animal studies^(^^35^^–^^37^^)^. However, studies investigating the effects of krill oil on serum cholesterol have reported conflicting results^(^^7^^)^.

In the present study, the expressions of *ACADVL*, *CPT1A* and *CPT2*, all genes encoding proteins involved in mitochondrial β-oxidation, were down-regulated in the krill group. Up-regulation of the β-oxidation pathway is suggested to be one of the mechanisms by which *n*-3 fatty acids reduce serum TAG^(^^45^^)^, making this finding somewhat counterintuitive. However, the change in levels of serum TAG was not significant in this study^(^^18^^)^, and it is possible that the effects on gene expression would have been different if a reduction in TAG had been seen. In addition, hepatic expression of *Acadvl*, *Cpt1a* and *Cpt2* and other genes involved in β-oxidation has been shown to be down-regulated in mice fed krill oil^(^^35^^,^^37^^)^, supporting our results. Moreover, NEFA may affect the expression of genes encoding proteins involved in β-oxidation^(^^46^^)^; however, levels of NEFA were not measured in this study.

The expression of *SCD* was significantly up-regulated within the fish group and the change in *SCD* expression was significantly different between the three intervention groups. Correspondingly, Telle-Hansen *et al.*^(^^11^^)^ previously found a non-significant increase in *SCD* expression in PBMC following intake of salmon for 2 weeks. Although *SCD* expression increased in the fish group, there was no change in the 18 : 1*n*-9/18 : 0 ratio, a marker of SCD activity, in our study (data not shown), suggesting that the altered gene expression of *SCD* did not affect desaturase activity. Furthermore, *TNF* expression decreased within the fish group, and, interestingly, the change in *TNF* expression correlated with the change in vitamin D levels. Intake of vitamin D has been shown to reduce cytokine production by inhibiting NF-κB signalling^(^^47^^)^ and our findings hence suggest that intake of fish rich in vitamin D, such as salmon and mackerel, may have a beneficial effect on inflammation.

Krill oil altered the expression of more genes than intake of lean and fatty fish with a similar amount of *n*-3 fatty acids. This finding is supported by the study by Burri *et al.*^(^^35^^)^ who found that krill oil altered the expression of 4892 genes, while equal doses of EPA and DHA from fish oil altered the expression of only 192 genes. Although the weekly intake of *n*-3 fatty acids in the present study was similar in the fish group and krill group, the intake of EPA in the krill group was 3·1 g/week, while the fish group had an intake of 1·4 g/week. This resulted in a greater increase in plasma EPA in the krill group than in the fish group^(^^18^^)^. According to a study by Tsunoda *et al.*^(^^48^^)^, supplementation with EPA altered the expression of more genes than DHA. Thus, the higher EPA content in krill oil compared with in fish might explain why krill oil altered the expression of a higher number of genes.

Unexpectedly, the changes in gene expression were more similar in the krill group and the control group than in the fish group. Notably, seven of the thirteen genes that were significantly down-regulated in the krill group were also significantly down-regulated in the control group. Because the control oil used in the present study was a HOSO, participants in the control group had a MUFA intake of 3 g/d from the HOSO capsules. Thus, it is conceivable that the findings in the control group might be mediated by MUFA. This is supported by a study by Bouwens *et al.*^(^^49^^)^ who showed that intake of HOSO altered the gene expression in the same manner as fish oil, indicating that supplementation with MUFA and *n*-3 PUFA might have some similar effects. In line with our findings, they also reported that HOSO down-regulated inflammation-related genes^(^^49^^)^. Furthermore, a substudy of the Prevención con Dieta Mediterránea (PREDIMED) study showed that expression of CD40 in PBMC was down-regulated in the groups receiving a Mediterranean diet high in MUFA^(^^50^^)^. This is in line with the present study showing that CD40 expression decreased significantly within the control group, and that the change in CD40 expression was significantly different between the three intervention groups. Moreover, the HOSO had added astaxanthin in an equal concentration as in the krill oil; thus, astaxanthin may have mediated some of the similar effects observed in the krill group and the control group. Astaxanthin is shown to reduce nuclear translocation of NF-κB and thereby inhibit the expression of inflammatory genes^(^^51^^)^. Thus, intake of astaxanthin may be another mediator of the down-regulation of inflammation-related genes observed in the control and krill groups.

The lack of effect on circulating markers of inflammation and endothelial dysfunction in the present study might be due to a relatively low intake of *n*-3 fatty acids. Whereas the participants in the present study received between 4·0 and 4·6 g of marine *n*-3 fatty acids per week, doses of 2 g/d (equal to 14 g/week) have been reported to have anti-inflammatory effects. Fish oil has been shown to lower the level of inflammatory cytokines in rheumatoid arthritis patients and in mononuclear cells from healthy individuals upon treatment with endotoxin^(^^20^^)^. Hence, as we recruited healthy participants with a C-reactive protein < 10 mg/l, the lack of effect on cytokines and adhesion molecules might not be surprising.

Our study has some limitations. First, because of the small sample size, we did not adjust for multiple testing among the variables to lower the risk of type II errors. Thus, our findings need to be interpreted with some caution. We used PBMC as a model system to study effects on glucose and lipid metabolism. Preferably, we would have wanted to study these effects of the intervention in liver biopsies; however, this is not possible due to both practical and ethical reasons. Nonetheless, PBMC seem to reflect hepatic gene expression of genes in cholesterol metabolism^(^^52^^)^. Another challenge when using PBMC is that they include several different cell types, including monocytes and lymphocytes. Therefore, a change in gene expression might reflect a change in cellular composition of the PBMC pool. However, we did not see any differences in the leucocyte count of different cell types from baseline to the end of the study (data not shown). Finally, gene expression analysis does not provide information on phenotype; hence, analysis of protein level and activity would give a more complete understanding of the effects of the intervention than only analysis of mRNA expression. Nevertheless, our study is strengthened by the randomised controlled study design and the high compliance of the participants.

In conclusion, analysis of PBMC gene expression after intake of krill oil, HOSO and lean and fatty fish revealed that intake of krill oil and HOSO with added astaxanthin regulated more genes than intake of fish. Furthermore, krill oil down-regulated the expression of several genes, such as genes involved in glucose and cholesterol metabolism as well as β-oxidation, while HOSO down-regulated several genes, including genes involved in inflammation. However, as we analysed a limited number of genes in each pathway, studies investigating the whole-genome transcriptome after intake of krill oil and lean and fatty fish are needed for a more comprehensive understanding of their effects on human health.

## Supplementary material

For supplementary material accompanying this paper visit https://doi.org/10.1017/jns.2018.2 click here to view supplementary material
