# Supplementary material for: Effects of fish and krill oil on gene expression in peripheral blood mononuclear cells and circulating markers of inflammation: a randomised controlled trial
Source: J Nutr Sci. 2018 Mar 21;7:e10. doi: 10.1017/jns.2018.2 (PMC5869279; doi:10.1017/jns.2018.2)
Supplement: Supplementary file 1 [file S2048679018000022sup001.zip › JNS 1800002 Supp Appendix 3.docx]

**Supplementary Appendix S3.** Correlation, analysed with Pearson’s correlation, between the change in mRNA expression (log ratio) and the change in plasma levels of *n*-3 fatty acids (weight % EPA + DPA + DHA)

| Gene | *P* | *r* |
| --- | --- | --- |
| *ABCA1* | 0.37 | 0.2 |
| *ABCG1* | 0.25 | 0.2 |
| *ACACA* | 0.43 | -0.1 |
| *ACADVL* | 0.97 | 0.0 |
| *ACOX1* | 0.34 | 0.2 |
| *ACSL5* | 0.57 | 0.1 |
| *CCL2* | 0.89 | 0.0 |
| *CCR2* | 0.53 | 0.1 |
| *CD36* | 0.84 | 0.0 |
| *CD40* | 0.14 | 0.2 |
| *CETP* | 0.47 | -0.1 |
| *CPT1A* | 0.78 | 0.0 |
| *CPT1B* | 0.90 | 0.0 |
| *CPT2* | 0.74 | -0.1 |
| *FADS1* | 0.99 | 0.0 |
| *FADS2* | 0.13 | -0.3 |
| *FASN* | 0.63 | -0.1 |
| *GPAM* | 0.69 | 0.1 |
| *HMGCR* | 0.92 | 0.0 |
| *HMGCS1* | 0.23 | -0.2 |
| *LCAT* | 0.79 | 0.0 |
| *LIPE* | 0.52 | 0.1 |
| *LPL* | 0.79 | 0.0 |
| *NR1H3* | 0.70 | 0.1 |
| *PDK4* | 0.26 | -0.2 |
| *PIK3R1* | 0.90 | 0.0 |
| *PLA2G4A* | 0.84 | 0.0 |
| *PPARD* | 0.49 | 0.1 |
| *PPARGC1A* | **0.006** | -0.5 |
| *PPARG* | 0.41 | 0.1 |
| *SCD* | 0.82 | 0.0 |
| *SLC25A12* | 0.76 | 0.1 |
| *SREBF1* | **0.04** | 0.4 |
| *SREBF2* | 0.32 | -0.2 |
| *TLR2* | 0.10 | 0.3 |
| *TLR4* | 0.62 | -0.1 |
| *TNF* | 0.06 | 0.3 |
| *TNFRSF1A* | 0.92 | 0.0 |
| *TNFRSF1B* | 0.66 | -0.1 |
| *UCP2* | 0.71 | -0.1 |
