# Supplementary material for: Effects of fish and krill oil on gene expression in peripheral blood mononuclear cells and circulating markers of inflammation: a randomised controlled trial
Source: J Nutr Sci. 2018 Mar 21;7:e10. doi: 10.1017/jns.2018.2 (PMC5869279; doi:10.1017/jns.2018.2)
Supplement: Supplementary file 1 [file S2048679018000022sup001.zip › JNS 1800002 Supp Appendix 2.docx]

**Supplementary Appendix S2.** Correlation, analysed with Pearson’s correlation, between the change in mRNA expression (log ratio) of inflammation-related genes and the change in vitamin D levels

| Gene | *P* | *r* |
| --- | --- | --- |
| *TNFRSF1B* | 0.45 | -0.1 |
| *CCL2* | 0.44 | -0.1 |
| *CCR2* | 0.83 | 0.0 |
| *TLR4* | 0.53 | 0.1 |
| *TNF* | **0.05** | -0.3 |
| *PIK3R1* | 0.80 | 0.0 |
| *TLR2* | 0.58 | -0.1 |
| *CD40* | 0.29 | -0.2 |
| *TNFRSF1A* | 0.67 | 0.1 |
