# Supplementary material for: Effects of fish and krill oil on gene expression in peripheral blood mononuclear cells and circulating markers of inflammation: a randomised controlled trial
Source: J Nutr Sci. 2018 Mar 21;7:e10. doi: 10.1017/jns.2018.2 (PMC5869279; doi:10.1017/jns.2018.2)
Supplement: Supplementary file 1 [file S2048679018000022sup001.zip › JNS 1800002 Supp Appendix 1.docx]

**Supplementary Appendix S1.** mRNA level at baseline, given as ∆Ct (normalized for *TBP*) and the change from baseline to end of study, given as ∆∆Ct (log ratio). The difference from baseline to end of study within each group is tested with a paired t-test, and the difference in change in gene expression between the groups is tested with one-way ANOVA. Significant findings were further tested with *post hoc* pairwise *t*-tests with Bonferroni corrected *P*-values and significant pairwise comparisons are indicated with symbols.

|  |  |  | ΔCt | | ΔΔCt | |  |  |
| --- | --- | --- | --- | --- | --- | --- | --- | --- |
| Gene | Group | n | Mean | SD | Mean | SD | *P** | *P*** |
| *ABCA1* | Fish | 11 | -3.25 | 0.62 | 0.10 | 0.49 | 0.52 | **0.03†** |
|  | Control | 12 | -2.92 | 0.72 | -0.61 | 0.60 | **0.005** |  |
|  | Krill | 12 | -2.81 | 0.41 | -0.25 | 0.69 | 0.24 |  |
| *ABCG1* | Fish | 11 | -2.95 | 0.50 | -0.01 | 0.60 | 0.95 | 0.12 |
|  | Control | 10 | -2.62 | 0.56 | -0.38 | 0.41 | **0.02** |  |
|  | Krill | 12 | -2.79 | 0.42 | 0.08 | 0.53 | 0.60 |  |
| *ACACA* | Fish | 12 | -2.07 | 0.75 | 0.18 | 0.93 | 0.51 | 0.16 |
|  | Control | 12 | -1.83 | 0.27 | -0.29 | 0.47 | 0.06 |  |
|  | Krill | 12 | -1.76 | 0.37 | -0.33 | 0.63 | 0.10 |  |
| *ACADVL* | Fish | 11 | 3.07 | 0.31 | -0.23 | 0.42 | 0.10 | 0.88 |
|  | Control | 12 | 3.07 | 0.34 | -0.31 | 0.51 | 0.06 |  |
|  | Krill | 12 | 3.12 | 0.35 | -0.24 | 0.32 | **0.02** |  |
| *ACOX1* | Fish | 12 | 0.04 | 0.56 | -0.04 | 0.72 | 0.87 | 0.48 |
|  | Control | 12 | 0.15 | 0.33 | -0.24 | 0.42 | 0.07 |  |
|  | Krill | 11 | 0.11 | 0.39 | 0.01 | 0.38 | 0.90 |  |
| *ACSL5* | Fish | 12 | 0.17 | 0.59 | -0.01 | 0.78 | 0.95 | 0.22 |
|  | Control | 11 | 0.24 | 0.30 | -0.40 | 0.58 | **0.04** |  |
|  | Krill | 12 | 0.02 | 0.26 | -0.03 | 0.29 | 0.76 |  |
| *CCL2* | Fish | 12 | -6.85 | 1.11 | 0.14 | 0.90 | 0.61 | 0.36 |
|  | Control | 11 | -6.32 | 1.54 | -0.57 | 1.57 | 0.25 |  |
|  | Krill | 10 | -6.94 | 1.59 | -0.32 | 0.99 | 0.33 |  |
| *CCR2* | Fish | 12 | 2.28 | 0.59 | -0.06 | 0.64 | 0.76 | 0.23 |
|  | Control | 12 | 2.62 | 0.51 | -0.49 | 0.59 | **0.02** |  |
|  | Krill | 12 | 2.77 | 0.57 | -0.31 | 0.59 | 0.10 |  |
| *CD36* | Fish | 11 | 1.68 | 0.67 | -0.01 | 0.50 | 0.97 | 0.37 |
|  | Control | 12 | 1.78 | 0.56 | -0.32 | 0.59 | 0.09 |  |
|  | Krill | 12 | 1.97 | 0.53 | -0.29 | 0.63 | 0.14 |  |
| *CD40* | Fish | 12 | -1.05 | 0.47 | -0.08 | 0.77 | 0.71 | **0.04** |
|  | Control | 12 | -0.68 | 0.58 | -0.65 | 0.51 | **0.001** |  |
|  | Krill | 12 | -0.89 | 0.40 | 0.00 | 0.67 | 0.99 |  |
| *CETP* | Fish | 11 | -5.60 | 0.40 | -0.19 | 0.60 | 0.32 | 0.79 |
|  | Control | 12 | -4.88 | 0.89 | -0.40 | 0.65 | 0.06 |  |
|  | Krill | 11 | -5.42 | 0.95 | -0.34 | 0.94 | 0.25 |  |
| *CPT1A* | Fish | 12 | 1.15 | 0.39 | -0.28 | 0.55 | 0.11 | 0.92 |
|  | Control | 12 | 1.17 | 0.33 | -0.32 | 0.46 | **0.04** |  |
|  | Krill | 12 | 1.08 | 0.38 | -0.36 | 0.39 | **0.009** |  |
| *CPT1B* | Fish | 12 | -4.34 | 0.54 | 0.16 | 0.62 | 0.38 | 0.47 |
|  | Control | 11 | -4.30 | 0.72 | -0.04 | 0.62 | 0.84 |  |
|  | Krill | 12 | -4.47 | 0.40 | 0.29 | 0.66 | 0.16 |  |
| *CPT2* | Fish | 12 | -1.05 | 0.55 | -0.15 | 0.51 | 0.33 | 0.50 |
|  | Control | 12 | -0.91 | 0.40 | -0.39 | 0.67 | 0.07 |  |
|  | Krill | 12 | -0.96 | 0.44 | -0.38 | 0.45 | **0.01** |  |
| *FADS1* | Fish | 12 | -0.47 | 0.73 | -0.16 | 0.72 | 0.45 | 0.93 |
|  | Control | 12 | -0.47 | 0.46 | -0.25 | 0.56 | 0.15 |  |
|  | Krill | 12 | -0.54 | 0.47 | -0.21 | 0.44 | 0.13 |  |
| *FADS2* | Fish | 11 | -0.58 | 1.11 | -0.05 | 0.76 | 0.84 | 0.23 |
|  | Control | 11 | -0.70 | 0.90 | -0.15 | 0.56 | 0.40 |  |
|  | Krill | 12 | -0.71 | 0.73 | -0.47 | 0.49 | **0.006** |  |
| *FASN* | Fish | 12 | 0.77 | 0.53 | 0.02 | 0.84 | 0.94 | 0.47 |
|  | Control | 12 | 0.82 | 0.50 | -0.28 | 0.49 | 0.08 |  |
|  | Krill | 12 | 0.93 | 0.30 | -0.18 | 0.31 | 0.07 |  |
| *GPAM* | Fish | 12 | -1.86 | 0.60 | 0.22 | 0.62 | 0.24 | 0.16 |
|  | Control | 12 | -1.59 | 0.32 | -0.25 | 0.68 | 0.23 |  |
|  | Krill | 12 | -1.75 | 0.25 | -0.03 | 0.44 | 0.81 |  |
| *HMGCR* | Fish | 12 | 0.36 | 0.36 | -0.08 | 0.43 | 0.52 | 0.06 |
|  | Control | 12 | 0.55 | 0.29 | -0.47 | 0.52 | **0.01** |  |
|  | Krill | 12 | 0.57 | 0.30 | -0.47 | 0.38 | **0.001** |  |
| *HMGCS1* | Fish | 12 | -1.66 | 0.47 | -0.16 | 0.68 | 0.42 | 0.48 |
|  | Control | 12 | -1.56 | 0.26 | -0.42 | 0.41 | **0.005** |  |
|  | Krill | 12 | -1.53 | 0.31 | -0.37 | 0.49 | **0.02** |  |
| *LCAT* | Fish | 12 | -2.27 | 0.31 | -0.04 | 0.47 | 0.79 | 0.21 |
|  | Control | 12 | -2.27 | 0.26 | -0.29 | 0.46 | **0.05** |  |
|  | Krill | 12 | -2.30 | 0.32 | 0.01 | 0.41 | 0.90 |  |
| *LIPE* | Fish | 12 | -1.25 | 0.54 | -0.09 | 0.59 | 0.59 | 0.17 |
|  | Control | 12 | -1.15 | 0.35 | -0.42 | 0.64 | **0.04** |  |
|  | Krill | 12 | -1.33 | 0.19 | -0.01 | 0.39 | 0.90 |  |
| *LPL* | Fish | 12 | -5.84 | 0.73 | -0.09 | 0.67 | 0.66 | 0.62 |
|  | Control | 12 | -5.86 | 1.03 | 0.00 | 0.93 | 0.99 |  |
|  | Krill | 12 | -5.58 | 0.89 | -0.33 | 0.89 | 0.23 |  |
| *NR1H3* | Fish | 12 | -2.69 | 0.41 | -0.13 | 0.50 | 0.38 | 0.29 |
|  | Control | 12 | -2.51 | 0.32 | -0.41 | 0.43 | **0.007** |  |
|  | Krill | 12 | -2.62 | 0.38 | -0.28 | 0.34 | **0.02** |  |
| *PDK4* | Fish | 12 | 0.85 | 0.56 | -0.04 | 0.73 | 0.86 | 0.28 |
|  | Control | 11 | 0.75 | 0.68 | 0.03 | 0.48 | 0.85 |  |
|  | Krill | 12 | 1.05 | 0.63 | -0.34 | 0.49 | **0.04** |  |
| *PIK3R1* | Fish | 12 | 2.55 | 0.61 | 0.13 | 0.98 | 0.65 | 0.17 |
|  | Control | 12 | 2.78 | 0.35 | -0.39 | 0.43 | **0.01** |  |
|  | Krill | 12 | 2.58 | 0.24 | -0.12 | 0.41 | 0.32 |  |
| *PLA2G4A* | Fish | 12 | -2.65 | 0.63 | -0.08 | 0.76 | 0.71 | 0.83 |
|  | Control | 12 | -2.40 | 0.64 | -0.29 | 0.84 | 0.26 |  |
|  | Krill | 12 | -2.26 | 0.70 | -0.24 | 0.96 | 0.41 |  |
| *PPARD* | Fish | 12 | 0.71 | 0.41 | -0.25 | 0.62 | 0.19 | 0.56 |
|  | Control | 12 | 0.75 | 0.33 | -0.38 | 0.58 | **0.05** |  |
|  | Krill | 12 | 0.71 | 0.33 | -0.13 | 0.41 | 0.29 |  |
| *PPARG* | Fish | 12 | -5.58 | 0.89 | 0.17 | 1.17 | 0.62 | 0.17 |
|  | Control | 12 | -5.07 | 0.48 | -0.45 | 0.80 | 0.08 |  |
|  | Krill | 12 | -5.63 | 0.70 | 0.31 | 1.07 | 0.34 |  |
| *PPARGC1A* | Fish | 12 | -7.05 | 1.45 | -0.10 | 0.91 | 0.72 | **0.01‡** |
|  | Control | 10 | -7.71 | 1.76 | 0.38 | 1.09 | 0.29 |  |
|  | Krill | 12 | -6.43 | 0.96 | -0.91 | 0.98 | **0.008** |  |
| *SCD* | Fish | 11 | -2.57 | 0.61 | 0.44 | 0.63 | **0.05** | **0.05** |
|  | Control | 12 | -2.46 | 0.45 | -0.16 | 0.61 | 0.37 |  |
|  | Krill | 12 | -2.24 | 0.57 | -0.08 | 0.59 | 0.65 |  |
| *SLC25A12* | Fish | 12 | -0.22 | 0.55 | -0.48 | 0.76 | **0.05** | 0.67 |
|  | Control | 12 | -0.22 | 0.29 | -0.55 | 0.60 | **0.008** |  |
|  | Krill | 12 | -0.29 | 0.37 | -0.34 | 0.37 | **0.01** |  |
| *SREBF1* | Fish | 11 | -0.33 | 0.53 | -0.06 | 0.51 | 0.68 | 0.21 |
|  | Control | 9 | -0.20 | 0.33 | -0.40 | 0.53 | 0.06 |  |
|  | Krill | 12 | -0.19 | 0.37 | 0.00 | 0.53 | 0.98 |  |
| *SREBF2* | Fish | 12 | 1.81 | 0.51 | -0.16 | 0.66 | 0.43 | 0.70 |
|  | Control | 12 | 1.68 | 0.24 | -0.18 | 0.48 | 0.22 |  |
|  | Krill | 12 | 1.78 | 0.31 | -0.32 | 0.38 | **0.01** |  |
| *TLR2* | Fish | 12 | 0.09 | 0.53 | 0.11 | 0.50 | 0.48 | 0.13 |
|  | Control | 12 | 0.37 | 0.58 | -0.35 | 0.68 | 0.10 |  |
|  | Krill | 12 | 0.34 | 0.52 | 0.00 | 0.52 | 0.98 |  |
| *TLR4* | Fish | 12 | 0.11 | 0.61 | 0.05 | 0.62 | 0.77 | 0.37 |
|  | Control | 12 | 0.21 | 0.59 | -0.21 | 0.45 | 0.13 |  |
|  | Krill | 12 | 0.44 | 0.78 | -0.26 | 0.66 | 0.20 |  |
| *TNF* | Fish | 12 | -1.21 | 0.54 | -0.44 | 0.60 | **0.03** | 0.30 |
|  | Control | 12 | -0.81 | 1.44 | -0.83 | 1.50 | 0.08 |  |
|  | Krill | 12 | -1.35 | 0.37 | -0.21 | 0.49 | 0.16 |  |
| *TNFRSF1A* | Fish | 12 | 0.70 | 0.62 | -0.02 | 0.93 | 0.93 | 0.96 |
|  | Control | 12 | 0.69 | 0.39 | -0.08 | 0.68 | 0.69 |  |
|  | Krill | 12 | 0.84 | 0.54 | 0.01 | 0.61 | 0.95 |  |
| *TNFRSF1B* | Fish | 12 | 4.15 | 0.49 | -0.15 | 0.51 | 0.33 | 0.47 |
|  | Control | 12 | 4.29 | 0.53 | -0.35 | 0.53 | **0.04** |  |
|  | Krill | 12 | 4.38 | 0.47 | -0.38 | 0.44 | **0.01** |  |
| *UCP2* | Fish | 12 | 5.00 | 0.51 | -0.42 | 0.67 | **0.05** | 0.53 |
|  | Control | 12 | 5.09 | 0.47 | -0.64 | 0.55 | **0.002** |  |
|  | Krill | 11 | 5.26 | 0.61 | -0.68 | 0.57 | **0.002** |  |

* *P*-value from paired *t*-test of within group change.

***P*-value from one-way ANOVA to test difference in change in gene expression between the groups.

† Significantly different between fish group and control group (*P*<0.05).

‡ Significantly different between krill group and control group (*P*<0.05).
